# Supplementary figures and images for: Unprecedented female mutation bias in the aye-aye, a highly unusual lemur from Madagascar
Source: PLoS Biol. 2025 Feb 7;23(2):e3003015. doi: 10.1371/journal.pbio.3003015 (PMC11819580; doi:10.1371/journal.pbio.3003015)

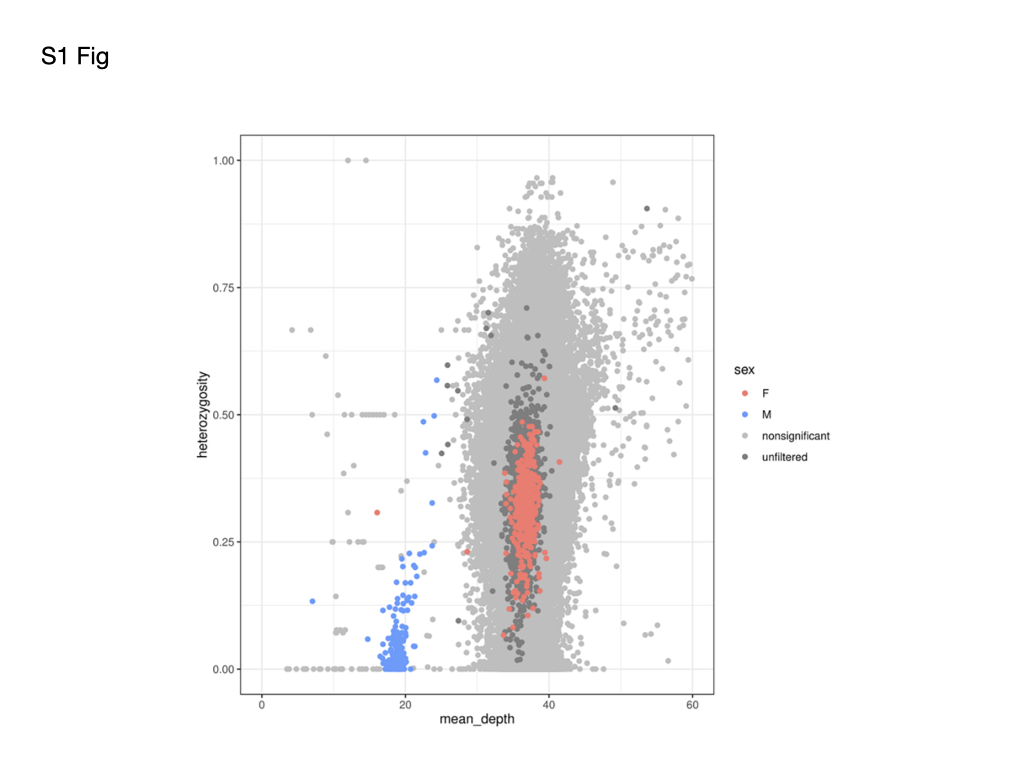

Supplement: S1 Fig — For each of the 6,289 contigs greater than 100 kilobases in length, the read-depth and heterozygosity in each individual was measured. Each contig is shown here represented by two points: one for the male mean and one for the female mean. Colored points represent contigs that have significantly (P < 0.05) different read-depth OR heterozygosity between the sexes AND that have <25X read-depth in males; these were inferred to be X-linked and were not used to identify mutations. Contigs with significant differences in read-depth or heterozygosity but that did not have read-depth <25X in males are in darker gray (“unfiltered”). No individuals were observed whose assigned sex was discordant with their expected read-depth and heterozygosity. The data underlying this figure can be found in S1 Data. (JPEG) [file pbio.3003015.s001.jpeg]

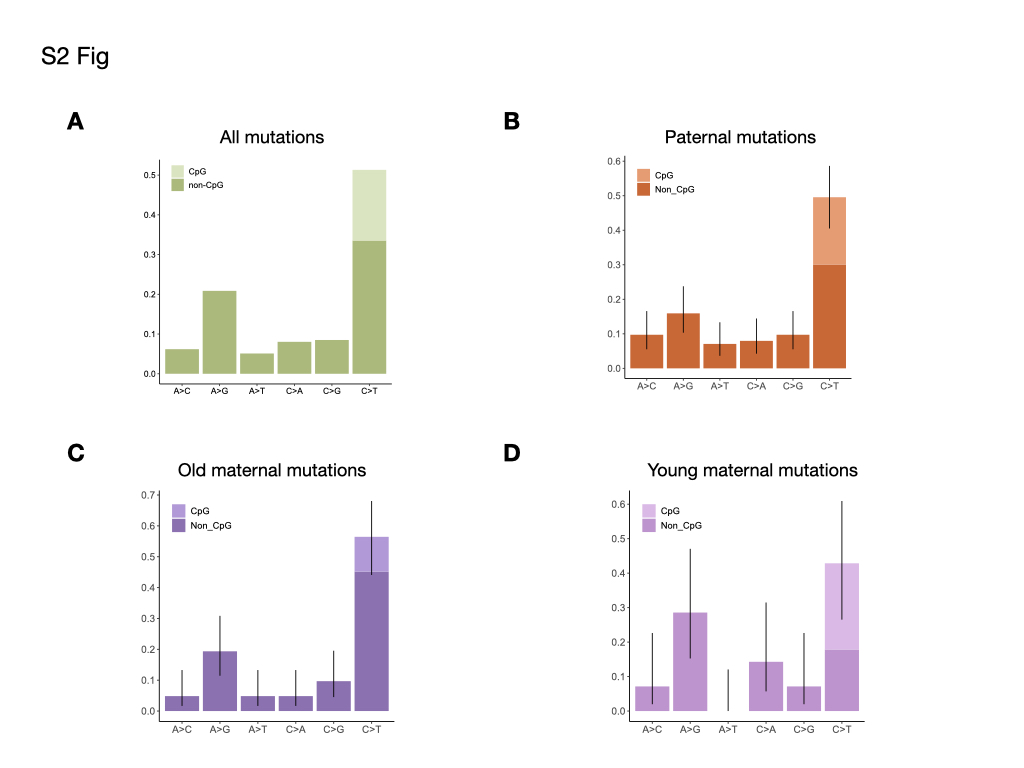

Supplement: S2 Fig — (A) The frequency of each mutation type is shown, among all 647 DNMs identified. (Mutation types represent their reverse-complement as well.) Mutations at CpG sites accounted for 17.7% of all mutations. (B) The frequency of all mutations assigned as coming from male parents. (C) The frequency of all mutations assigned as coming from the two female parents at the three oldest ages of birth. (D) The frequency of all mutations assigned as coming from all other female parents and births. The data underlying this figure can be found in S1 Data. (JPEG) [file pbio.3003015.s002.jpeg]

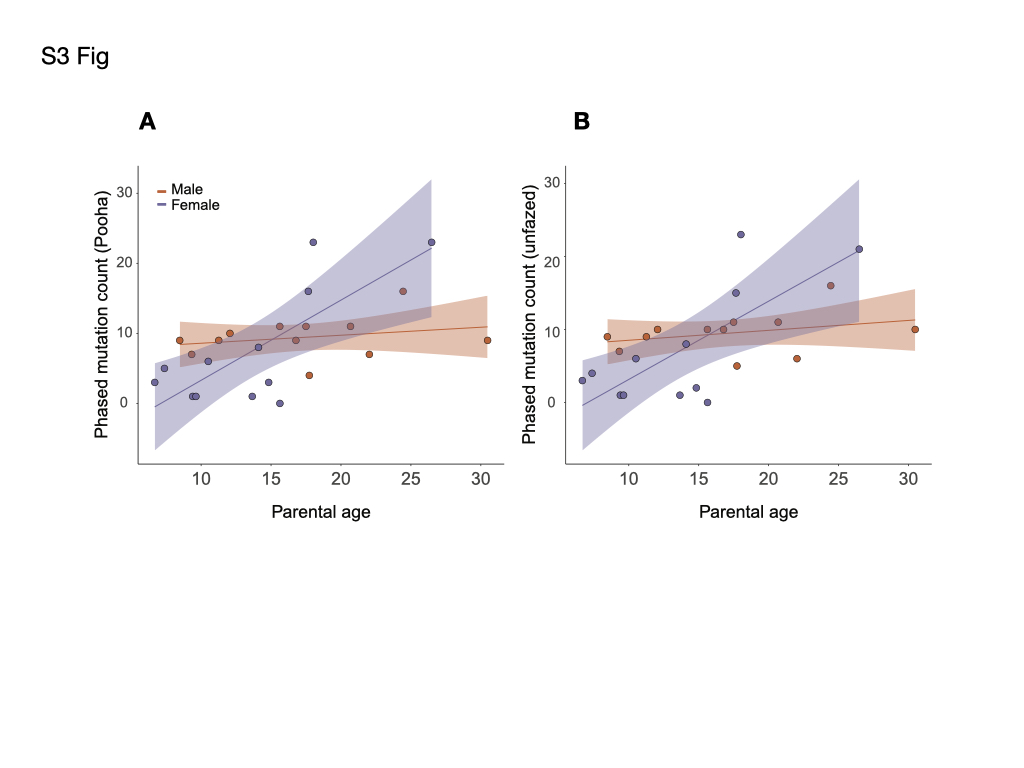

Supplement: S3 Fig — (A) This figure contains the same data as are represented in Fig 2A, again showing mutations assigned to parents using the software package POOHA. (B) This figure contains the same data as are represented in Fig 2A, but observed mutation counts are from assignments to parents using the software package Unfazed. Significance of coefficients for age effect in Poisson regression: Paternal P = 0.24, Maternal P = 0.04. The data underlying this figure can be found in S1 Data. (JPEG) [file pbio.3003015.s003.jpeg]

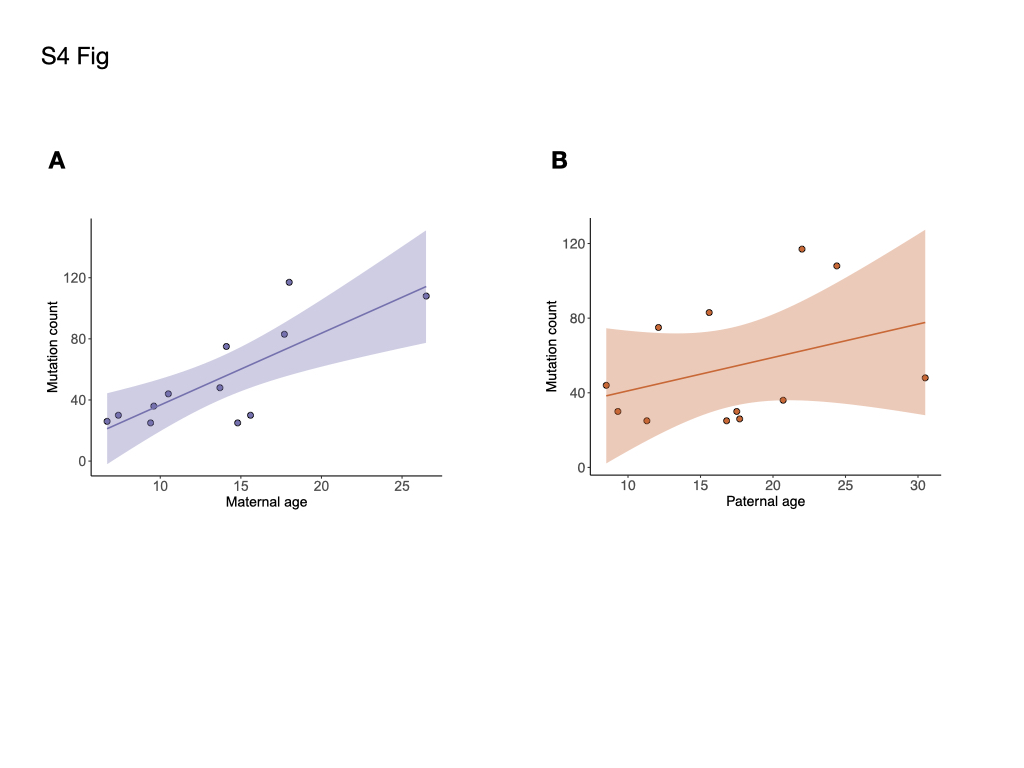

Supplement: S4 Fig — The data underlying this figure can be found in S1 Data. (JPEG) [file pbio.3003015.s004.jpeg]

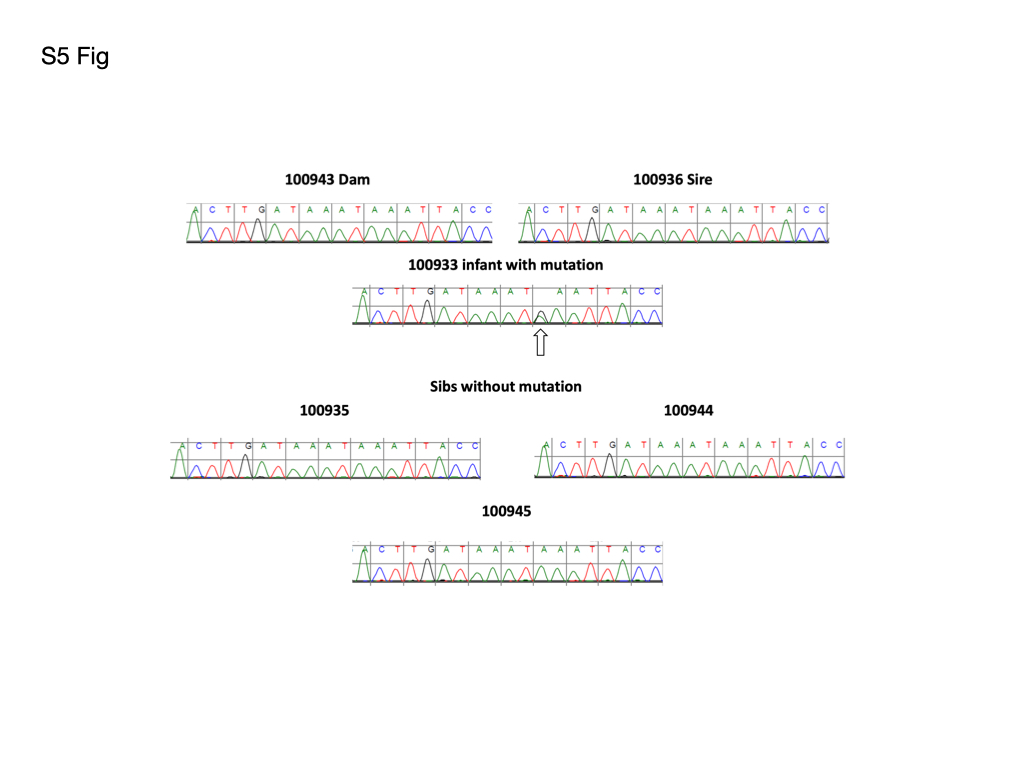

Supplement: S5 Fig — Electropherograms for the region around an inferred de novo mutation (indicated by an arrow in the proband) are shown for the family in which the mutation appeared. Only the proband is heterozygous at this position. ID numbers refer to Fig 1A. (JPEG) [file pbio.3003015.s005.jpeg]

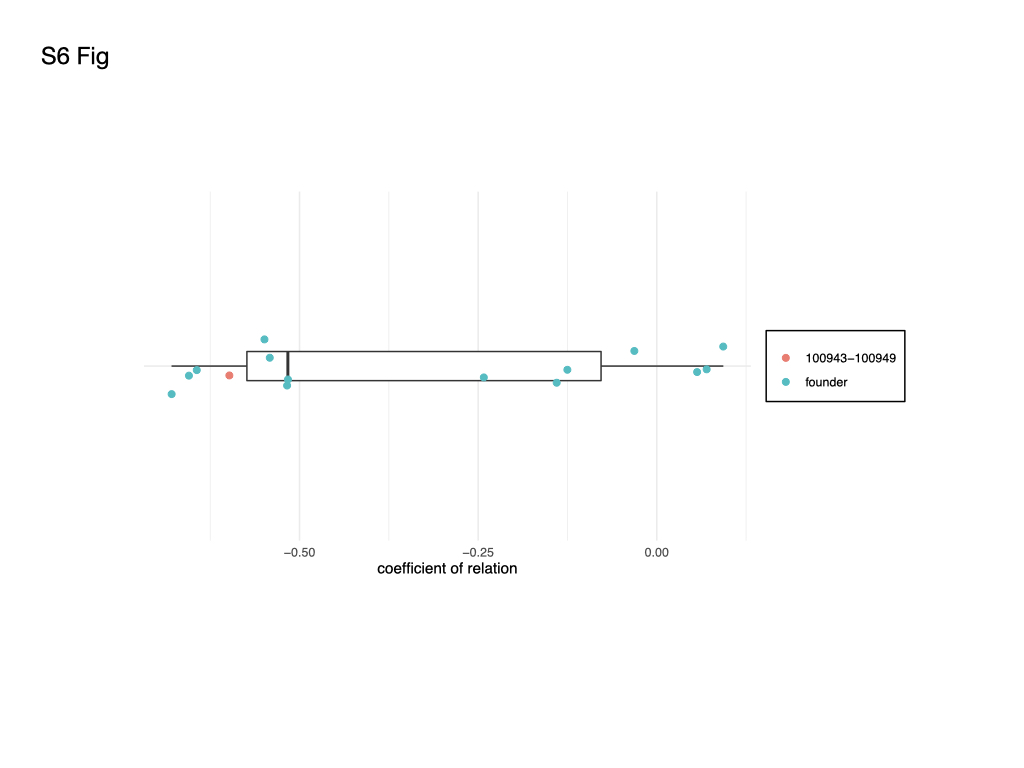

Supplement: S6 Fig — We estimated the coefficient of relatedness (CoR) between all pairs of founders based on their genotypes at a random sample of 100,000 variable sites. The CoR of the two mothers in our sample that had children at the oldest ages is shown as a red dot. Our estimate of the CoR follows the formula from Pedersen and Quinlan [69], in which a CoR of 0 or less indicates unrelated individuals. The data underlying this figure can be found in S1 Data. (JPEG) [file pbio.3003015.s006.jpeg]

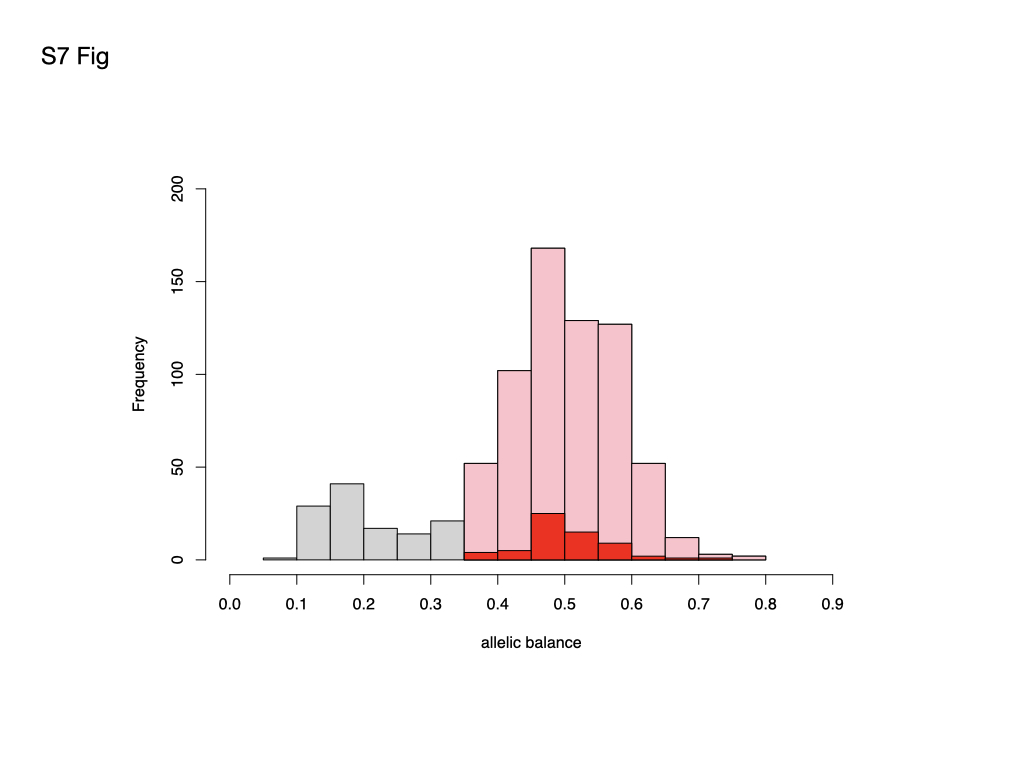

Supplement: S7 Fig — Distribution of allelic balance for candidate mutations is shown, highlighting the set of DNMs accepted after applying the threshold of 0.35. All candidates less than this threshold were discarded (gray), while all candidates greater than this threshold were kept as the final set (pink and red). The red bars indicate the allelic balance of all maternally transmitted mutations for the three oldest ages of maternal conception. The data underlying this figure can be found in S1 Data. (JPEG) [file pbio.3003015.s007.jpeg]

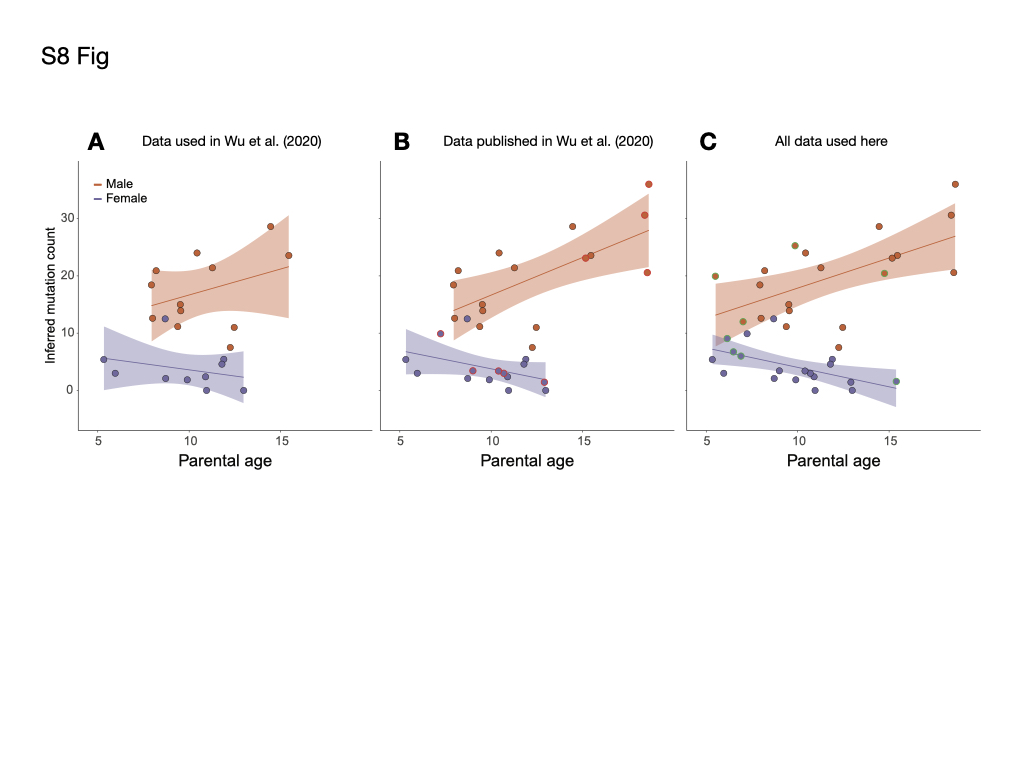

Supplement: S8 Fig — (A) Data from the same trios analyzed and used in Wu and colleagues [12]. (B) Data from all trios sequenced in Wu and colleagues [12]. The datapoints that have been added relative to panel A are highlighted in red. (C) All data used here, including all datapoints from panel B plus trios sequenced for the first time in this study (highlighted in green). The data underlying this figure can be found in S1 Data. (JPEG) [file pbio.3003015.s008.jpeg]
