# Supplementary material for: Unprecedented female mutation bias in the aye-aye, a highly unusual lemur from Madagascar
Source: PLoS Biol. 2025 Feb 7;23(2):e3003015. doi: 10.1371/journal.pbio.3003015 (PMC11819580; doi:10.1371/journal.pbio.3003015)
Supplement: S2 Table — Statistics on single-nucleotide variants from aye-ayes in our sample. (DOCX) [file pbio.3003015.s011.docx]

**Supplementary Table 2**

| ID | sex | ref hom | nonref hom | hets | missing | n_variants | ref_length | "heterozygosity" | Mean depth |
| --- | --- | --- | --- | --- | --- | --- | --- | --- | --- |
| 100933 | M | 1999185 | 566349 | 1738962 | 2221 | 4306717 | 2383586330 | 0.00073 | 39.8 |
| 100934 | M | 1864973 | 862371 | 1576435 | 2688 | 4306467 | 2383586330 | 0.00066 | 40.2 |
| 100935 | M | 2006593 | 560058 | 1737763 | 2368 | 4306782 | 2383586330 | 0.00073 | 38.5 |
| 100936 | M | 1929485 | 814068 | 1559979 | 3017 | 4306549 | 2383586330 | 0.00065 | 39.1 |
| 100937 | M | 2154212 | 716880 | 1433001 | 2641 | 4306734 | 2383586330 | 0.00060 | 40.0 |
| 100938 | F | 1939483 | 719514 | 1643769 | 3879 | 4306645 | 2383586330 | 0.00069 | 39.6 |
| 100939 | F | 2250913 | 488915 | 1563618 | 3454 | 4306900 | 2383586330 | 0.00066 | 43.3 |
| 100940 | F | 2193454 | 325673 | 1784490 | 3223 | 4306840 | 2383586330 | 0.00075 | 40.7 |
| 100941 | F | 2047290 | 581677 | 1674254 | 3662 | 4306883 | 2383586330 | 0.00070 | 37.6 |
| 100942 | F | 2202235 | 529490 | 1571645 | 3531 | 4306901 | 2383586330 | 0.00066 | 40.6 |
| *100943 | F | 2317359 | 651059 | 1334194 | 4264 | 4306876 | 2383586330 | 0.00056 | 44.6 |
| 100944 | F | 1963793 | 544010 | 1795270 | 3626 | 4306699 | 2383586330 | 0.00075 | 39.3 |
| 100945 | F | 1975336 | 533202 | 1794522 | 3751 | 4306811 | 2383586330 | 0.00075 | 40.5 |
| 100946 | F | 2024807 | 646704 | 1631686 | 3564 | 4306761 | 2383586330 | 0.00068 | 42.3 |
| 100947 | F | 1888651 | 886506 | 1527495 | 3802 | 4306454 | 2383586330 | 0.00064 | 45.6 |
| 100948 | F | 2676474 | 304925 | 1322696 | 3275 | 4307370 | 2383586330 | 0.00055 | 42.1 |
| *100949 | F | 1889472 | 874969 | 1538061 | 3969 | 4306471 | 2383586330 | 0.00065 | 39.8 |
| 100950 | F | 2191654 | 327371 | 1784563 | 3253 | 4306841 | 2383586330 | 0.00075 | 39.7 |

* focal mothers

“heterozygosity” = hets / (ref_length – missing)

ref_length calculated as total length from 397 contigs that were at least 100kb
