# Supplementary material for: Unprecedented female mutation bias in the aye-aye, a highly unusual lemur from Madagascar
Source: PLoS Biol. 2025 Feb 7;23(2):e3003015. doi: 10.1371/journal.pbio.3003015 (PMC11819580; doi:10.1371/journal.pbio.3003015)
Supplement: Attachment — Submitted filename: Response_v2a.docx [file pbio.3003015.s017.docx]

Dear Roli,

Thank you very much for the constructive reviews. Below we provide detailed answers and/or detailed descriptions of the changes we made to our manuscript. In summary, we have made the following changes:

- We are much clearer in multiple places in the paper that our results are driven by three offspring. We have also carried out additional analyses to exclude possible technical or biological differences in these offspring and their mothers.
- We provide a detailed analysis of mutational spectra, as well as other requested analyses.
- We clarify that de Manuel et al. (2022) did not collect pedigree data, but that our phylogenetic estimate of sex bias matches their phylogenetic estimate very well.

While we did not carry out additional sequencing, we have carried out an additional analysis demonstrating that female-biased mutations are transmitted to a third generation and are therefore not simply somatic errors.

We hope that these responses and our changes have addressed all of the reviewers’ concerns.

Thank you,

Matt (for authors)

Dear Matt,

Thank you for your patience while your manuscript "Unprecedented female mutation bias in aye-ayes" was peer-reviewed at PLOS Biology. It has now been evaluated by the PLOS Biology editors, an Academic Editor with relevant expertise, and by three independent reviewers. 

You'll see that reviewer #1 calls the findings “striking” and “intriguing,” but notes that only 3 offspring have a maternal bias and asks whether maternal age should be factored in. Reviewer #2 is also very positive, and notes the same n=3 issue, asking you to flag this more prominently and to rule out a shared mutator phenotype. S/he also asks you to analyse mutational spectra, plus a few other analyses and clarificatory questions. Reviewer #3 asks explicitly about the apparent disparity with de Manuel et al., wants some discussion of mechanism, requests several further analyses, additional sequence validation, and (like rev #2) asks about mutational spectra.

In light of the reviews, which you will find at the end of this email, we would like to invite you to revise the work to thoroughly address the reviewers' reports.

Given the extent of revision needed, we cannot make a decision about publication until we have seen the revised manuscript and your response to the reviewers' comments. Your revised manuscript is likely to be sent for further evaluation by all or a subset of the reviewers.

********************************************************************************

REVIEWERS' COMMENTS:


Reviewer #1: 

Wang et al reveal a striking new result in the field of germline mutation rates and sex bias in mammalian genomes by finding evidence for female mutation bias in aye-ayes.

They use whole-genome sequencing in multiple pedigrees and employing standard methods to call and phase mutations. As a control, they use WGS on baboon pedigrees.

This is a very intriguing result, and I would ask the authors to address a few questions and concerns.

1. Looking closely at Figure 1A and Table 1, only three aye-aye offspring have a maternal bias in mutation bias. They are 100947 with 7 paternal, 23 maternal; 100933 with 16 paternal and 23 maternal; 100945 with 11 paternal and 16 maternal. They other 9 offspring in the study have a paternal bias and that bias is often quite strong (9:1 in 100938, 11:1 in 100941, 9:3 in 100950, 11:0 in 100942, and 9:1 in 100939). Thus alpha is consistent with a paternal bias in the majority of the offspring. What do the authors make of this? Moreover, for two of the 3 offspring with a maternal bias, they are in a family where two other offspring show a slight paternal bias. To me, it looks like maternal bias really only takes effect when mothers are older and perhaps also older than the father, which is the case for two of the three offspring with a maternal bias. This advanced maternal age does not seem to be the case for the baboons and it is rarely the case in human. I would ask that the
authors consider this as a confounder.

These are good points, and we should have been clearer in the original submission that only three offspring have a maternal bias. We have now updated the text to make this very clear, both in the Results (lines 237-258) and in the Discussion (lines 414-429). The new paragraph in the Results begins:

“It is notable that the female bias we observe in aye-ayes is driven by three offspring (100947, 100933, and 100945) arising from only two different mothers (100949 and 100943). Indeed, if we ignore these three offspring, the paternal contribution to *de novo* mutations is 74% (equivalent to α=2.82), within the range of 70-80% reported for most mammals (de Manuel et al. 2022; Bergeron et al. 2023). Although these are the oldest ages at conception of any mothers in our dataset, we looked for any further technical differences between these two mothers and other individuals that might explain the patterns we observe...”

We also earlier report explicitly the overall value of alpha (1.26) which is slightly male-biased, but much lower than values observed in other mammals except gray mouse lemur (lines 193-195).

While it is true that these three aye-aye mothers are older than any baboon mothers studied here, there are both older macaque and human mothers. There is no sign of any female bias in either of these species at advanced maternal ages. Nevertheless, we now mention all of these things as caveats in the Discussion (lines 423-429).

2. Since the effect is only seen in 1/2 of the offspring for one family, none of the offspring for another family, and 1/1 offspring in the third family, is it possible that sample swaps happened for subset?

We re-checked these relationships: they all appear to be correct. Incorrect pairings of parents and offspring results in many hundreds of thousands of apparent “mutations” as there are many alleles in the offspring that are not present in the assigned parents.

3. Could genome-wide differences in depth between mother and father genomes bias the assignment of DNMs to one sex versus the other? That is, allelic dropout in the father?

This is a good point. We now report read-depth for all individuals (new Supplementary Table 2): there is no consistent difference between the mothers and fathers or in the three offspring with more maternal mutations (lines 247-249).


Reviewer #2:

This paper presents a striking example of female-biased mutation, which is more-or-less accurately described as an unprecedented finding. This finding could be viewed as preliminary due to the study's small sample size (just two females and three offspring drive the result). However, publication is justified in my view since 1) larger studies of nonhuman primate mutagenesis are rare, and 2) the paper's data provide important information about where future studies of primate mutagenesis might want to focus their efforts and resources. Below, I suggest a few small analyses that might shed light on the nature of this maternal age effect, and I also have a few suggestions for further improving the clarity and accuracy of the text.

Figures 1 and 2 make it fairly transparent that the finding of female mutation bias is driven by just three trio datapoints, two of which share the same mother. However, I didn't see this fact explicitly mentioned anywhere in the paper text, and it seems appropriate to discuss this along with the other discussion section caveats. It is striking that these are the only three trios where the mother is over the age of 15 years, and that gives me confidence that the result has a good chance of being a universal feature of aye-aye biology, but it's also possible that these two mothers are unusual in some way. They might have a mutator phenotype like the one described in the Stendahl, et al paper (though one that is age-dependent, unlike that phenotype) or they might be unusual in their ability to reproduce at an advanced age. The discussion section mentions that not much is known regarding aye-aye generation times in the wild, but is there any information regarding the proportion
of female aye-ayes who have successfully given birth to live young at ages of 15+ years within the Duke lemur facility? It would also be useful to clarify whether these mothers have a known genealogical relationship to each other, or whether they share an unusual amount of their genomes identical by descent.

As also mentioned by reviewer #1, this is an important point that we failed to bring forward in the original submission. We now explicitly discuss the number of offspring driving the female bias in the Results (lines 237-258) and Discussion (lines 414-429), including speculation about possible genetic variation in the mutation process (lines 434-440).

With regard to the ages of aye-aye mothers in captivity: researchers at the Duke Lemur Center have told us that all four of their founding females gave birth after the age of 15, some of them multiple times. We now mention this in the Discussion (lines 483-484).

We also now report relatedness among all parents in our study (lines 245-247 and new Supplementary Figure 6). In summary, the female parents that transmit high numbers of mutations are not related at all.

The analysis of aye-aye DNA repair gene substitutions was interesting, but it could be improved by the addition of some more context. Do the baboons and macaques have similar numbers of apparently deleterious DNA repair gene substitutions? Do the results change at all if we look at all DNA repair gene mutations that are common to the two mothers who had children at ages of 15+ years, rather than just DNA repair gene mutations that appear fixed in aye-ayes?

Unfortunately, we did not examine DNA repair substitutions in baboons or macaques, and are not confident that a comparison of just the number of substitutions between lineages would be informative.

However, as suggested by the reviewer, we have now looked for polymorphisms specific to the two mothers in question. As is now reported in the Results (lines 330-341), we found polymorphisms in and around four DNA repair genes that were heterozygous in the two mothers with children at 15+ years of age, but were homozygous reference in all other mothers. Polymorphisms in two of these genes (*BLM* and *UBE2A*) were also absent from all male fathers in the dataset. We discuss the implications of these results for the possibility of variation in mutational processes among aye-ayes in the Discussion (lines 434-440).

It would be straightforward and informative to look at whether the aye-aye maternal age effect has similar spectral characteristics to the maternal age effect observed in humans. For example, are the three advanced maternal age trios enriched for C>G mutations relative to the other aye-aye trios, as has been seen for human advanced maternal age? If so, is there any evidence for clustering of the additional C>G mutations into genomic hotspot regions that might also show elevated levels of C>G substitutions or polymorphisms? Does the dependence of mutation rate on maternal age fit an exponential curve significantly better than a linear regression line, as has been seen in some human studies but not others?

This was a good suggestion, and we now report separate mutation spectra for the older and younger mothers, as well as for all fathers (Supplementary Figure 2). We found absolutely no differences in the mutation spectrum among groups (reported on lines 206-207).

We also did not observe any particular clustering of mutations in older aye-aye mothers. We only observed one multinucleotide mutation, and this was in an individual whose mother was 7.4 years at conception. We now make this clear in the Results (line 137).

I actually did not share the authors' worry that the X:autosome substitution results appear to contradict the DNM results. It seems unlikely that reproduction at 15+ years is any more common in nature than it is in captivity, given that life expectancy is probably longer in captivity and nutrition is likely more consistent. If one were to average together the maternal and paternal mutation loads across all the trios in the dataset, it looks likely to be male biased. I think it could be informative to do that calculation explicitly and test whether it yields an expected alpha value that is similar to the one computed phylogenetically. It would also be useful to mention whether the de novo mutation rate and spectrum appears to be any different on the aye-aye X chromosome compared to the autosomes, given that such a spectral difference was previously detected in humans by Agarwal & Przeworski. Is there any power to detect whether the maternal bias found on the autosomes also
applies to the X?

We followed the reviewer’s suggestion and calculated alpha across all of our samples. The value (1.26) is not similar to the one from the X:autosome substitution results (now reported on lines 193-195). However, we agree that there is no guarantee that our samples have average ages of conception that reflect those in nature. To further examine this idea, we now report (lines 472-476) the average age of male and female parents that would have to exist in nature to give the observed alpha from phylogenetic data (8.97 years).

Similarly to most trio studies in non-humans (and most studies in humans, actually), we did not call mutations on the X chromosome. The complications of hemizygosity mean that accepted thresholds for accurately calling de novo mutations on this chromosome have not been worked out.

Minor comments:

I assume that the authors tried and failed to find an explanation within the Wu, et al. paper for why these additional trios were not included in their analysis, but an explanation would be worthwhile to include if possible. It would also be useful to explicitly test whether the distribution of paternal ages is responsible for the differences between the two papers' results, as presented as a conjecture in lines 164-65. I also noticed that the two papers find quite different maternal age effects—in the current paper, the effect is negative, while in the Wu, et al. paper, it is larger than the paternal age effect. Do age distributions also appear to explain this contradiction?

We have definitely looked for an explanation as to why these trios were not included, but have found none. To be more complete, we have added a new figure (Supplementary Figure 8) that shows the male and female mutation contributions for three sets of baboon data: A) the same data as was published and analyzed by Wu et al., B) all data published in Wu et al., and C) all data used here, with additional trios highlighted.

As explained in Wu et al., the positive effect they observe in baboon mothers is driven by an outlier point. In contrast, that individual does not have an unusual number of maternally inherited mutations in our analysis, which may be causing the difference. We now report this in the Results (lines 279-283). Therefore, minor differences between the papers may be in the mutation-calling and phasing steps (now mentioned in the Discussion, lines 389-392).

I was surprised to see that the paternal and maternal regression p values in line 186 were so different, given that the maternal and paternal ages in Table 1 appear correlated to my eyes. Given this correlation, I would have expected the highly significant maternal age effect on the total mutation load to create at least some appearance of a paternal age effect. Any idea why this is not the case?

To make these relationships clearer, we have added a new Supplementary Figure 4 (first mentioned on line 200). While the paternal and maternal ages are generally correlated, the distributions are different enough to allow us to accurately assess the effect of each independently.

Lines 353-54 state that the Stendahl, et al. macaque paper provided the first "clear evidence for genetic variation that only affected germline mutation in females." I don't think Stendahl et al. showed this—they demonstrated that their MBD4 allele had a maternal effect, but they didn't have the right data to test whether or not it also had a paternal effect.

This has been fixed.

The reason I say above that I "more-or-less" agree with the use of the term "unprecedented" is that the Stendahl paper does contain examples of trios where the maternal lineage is the origin of the majority of the mutation load. Also, the Wu, et al. paper finds a maternal age effect that is nominally larger than the paternal age effect. The manuscript even says in line 326 that their results "have some precedent." However, these examples are minor enough that I would say the title is justified.


Reviewer #3:

This study presents an interesting and unexpected discovery regarding the patterns of de novo mutation transmission in aye-ayes primates. In contrast to the well-established male mutation bias observed across mammals, where males typically transmit more mutations to their offspring than females, the authors find that in aye-ayes, the opposite occurs. Maternal age has a much stronger effect on mutation rates than paternal age, and older mothers transmit significantly more mutations than older fathers, which is unprecedented in primates.

The authors utilized whole-genome sequencing data from 12 aye-aye trios and 4 baboon trios, using the same computational and experimental approaches for consistency. While baboons, rhesus macaques, and humans showed the expected male-biased mutation rates, the aye-aye data revealed a female mutation bias, particularly with increasing maternal age. This result suggests that mutation bias is not a fixed trait in mammals and can evolve in closely related species. This work challenges long-held assumptions about sex-biased mutation rates in mammals and opens the door to further investigations in this space.

Overall, the finding is certainly novel, and if it holds true, it will be highly significant for the field and of great interest to the readers of the journal. However, given that sex bias in germline mutation is a well-studied subject, with consistent patterns reported across taxa, this study would benefit from further follow-up validations to strengthen the robustness of the results. These additional efforts would help ensure that the observed maternal mutation bias is representative of the aye-aye species, rather than a peculiarity of the sampled individuals. Below, I have listed some of my concerns that I hope will help improve the clarity and overall robustness of the findings.

1-	The trio data from aye-ayes was previously sequenced by de Manuel et al. (2022), and they reported a male-biased mutation pattern like that observed in other primates and most mammals. Given this, I'm unclear why these new results would suggest such a strikingly different outcome, especially with the strong maternal bias and pronounced age effect. If this is accurate, there must be some fundamental biological differences causing this maternal bias, which is quite unusual, particularly given that oogenesis in mammals does not involve post-birth cell divisions. While recent work in humans (Abascal et al., 2021; Moore et al., 2021) suggests that mutations can accumulate with age in post-mitotic tissues, the rate of mutation accumulation in the maternal germline here seems unexpectedly high. I am not sure if this is discussed in depth in the current manuscript - especially since the outcome from the phylogeny trees are quite opposite to their findings! 

We have re-checked de Manuel et al. (2022): it does not contain any data from aye-aye trios. All of their data on aye-ayes are from phylogenetic comparisons, and in fact our phylogenetic results (α=3.1) closely match theirs (α=2.95; now on lines 308-309).

Given the disagreement between pedigree and phylogenetic data, we have added a new estimate of the average age of aye-aye males and females at conception that would have to exist in nature to give the observed alpha from phylogenetic data (lines 472-476). Our estimate (8.97 years) helps to reconcile these different observations from pedigrees and phylogenies.

2-	I am unclear whether any of the 12 trios share maternal or paternal line. A clear analysis of relatedness within these trios is important for strengthening the significance of the findings. I suggest comparing SNPs between all 12 trios to clarify any genetic relationships by genotype.

This point was also raised by reviewer 2: the new Supplementary Figure 6 shows that the parents in the first generation of our pedigrees are relatively unrelated (reported on lines 244-247). The closest relationship among any pair of parents appears to be that of second cousins, but the two mothers that passed on the most mutations are completely unrelated to each other.

3-	Additionally, it would be helpful to provide more clarity on how the overall mutation burden in these 12 trios, after accounting for parental age effect, compares with similar primates or human. This comparison would give more context to the findings.

The new Supplementary Figure 4 (line 200) plots the total number of mutations in each child against either A) maternal age or B) paternal age. We agree that this helps to give our results more context.

4-	Although paternal bias is consistently reported across taxa, there have been cases of maternal bias, as cited by the authors. In those instances, the maternal line often carries a hypermutator phenotype, meaning that the mother has an elevated germline or somatic mutation burden due to genetic factors such as MBD4 or MUTYH mutations, which impair DNA replication or repair. The analysis here does not seem sufficient to exclude the possibility of a hypermutator effect, particularly given the suspicion that some of these trios might be closely or distantly related. With the ~30-40X whole-genome sequencing, it's likely insufficient to identify enough coding mutations, particularly if these mutations are mosaic. Perhaps high depth targeted or exon sequencing of maternal genomes would add more clarity that none of these mums have a hypermutator germline. 

This is an interesting set of hypotheses, but we believe the data exclude a simple hypermutator phenotype. For one of these mothers (100943) we have four total offspring: the higher number of mutations transmitted is only found in the animals born at the oldest maternal ages. If this individual had a mutator phenotype that resulted in excess mosaic mutations as well, these would have been observed in all offspring. We observed two mutations shared between two siblings of this mother, but they were not clearly assigned as coming from the mother. No other shared mutations were observed (all discussed on lines 419-423).

We address additional questions about heterozygosity below, but just for completeness: there is nothing unusual about the heterozygosity of these mothers compared to any other individuals (new Supplementary Table 2) and nothing unusual about their relatedness (new Supplementary Figure 6).

5-	Given the importance of the findings, I would strongly recommend validating these de novo mutations (DNMs) using deep targeted sequencing across all trios. This would add much-needed robustness to the conclusions. Although the Sanger sequencing is useful approach to exclude possibility of germline mutation, it is fairly shallow and won't be able to detect any possibility of mosaic mutations. 

Our results show that three offspring inherit a much larger number of mutations from two aye-aye mothers. We now make clear that we do not know the cellular or molecular mechanism for these additional mutations, and that there could be genetic variation for the mutation rate among parents sequenced here (lines 434-440).

However, the hypothesis of germline mosaicism predicts many shared mutations among offspring of such individuals, but only two shared mutations were identified (out of at least 53 mutations transmitted by this mother). Furthermore, germline mosaicism in the mothers would not invalidate our conclusions, but instead would simply have pointed to a possible mechanistic explanation for them, without further explaining results from male aye-ayes. We also observe no relationship in aye-ayes between paternal age and number of transmitted mutations, suggesting differences in both males and females of this species.

6-	Additionally, it would be useful to expand this work on other trios from other centres to ensure this finding can be generalised and exclude possibility of certain. Environmental exposures that may cause hypermutation in these female lines.

We have spent the better part of the last year asking primate centers and zoos around the world for appropriate specimens. They either have no trios with known ages of parents at birth, or only offspring from very young mothers, which would not have helped to test the patterns observed here.

7-	The observation of a negative maternal age correlation in baboons is unexpected. Although the authors achieved better phasing results in baboons, they did not seem to replicate the expected parental age effects seen in previous studies. While modest sample size might contribute to this, previous publications have not reported this negative correlation, raising concerns about potential confounders.

We now include the new Supplementary Figure 8, which shows the parental age effects for A) the original data used in Wu et al., B) all the data published in Wu et al., and C) all of our data and data from Wu et al. together. As can be seen, given our mutation-calling and phasing results, even the Wu et al. original data shows a very slight negative slope. We now discuss this in the paper, including the need for larger sample sizes (lines 279-283 and 389-393).

8-	Is there any bias in the depth of coverage between maternal and paternal samples? The figure shows variation in mean coverage, but I don't see whether this was accounted for in the regression analyses. This could potentially affect the results, particularly for phased mutations.

As also raised by reviewer #2, we now include the new Supplementary Table 2, which shows that there is no difference in depth of coverage between maternal and paternal samples (lines 247-249).

9-	There also seems to be variation in heterozygosity levels between maternal and paternal samples. This raises the question of whether some of the observed maternal bias might be influenced by inbreeding, where mothers contribute more to the progeny's genetic makeup.

It is not clear what difference in heterozygosity the reviewer is referring to, but nevertheless we now include the heterozygosity of each aye-aye individual in the new Supplementary Table 2. As can be seen (and as mentioned in the Results, lines 247-249) there is no difference in heterozygosity between maternal and paternal samples.

10-	It would be useful to explore whether there are differences in the mutation spectra between maternal and paternal lineages, as similar analyses have been conducted in previous studies (e.g., Rahbari et al. 2015 or Moore et al., 2021). Understanding if there are distinct mutational signatures would add depth to the findings.

This point was also raised by reviewer #2, and we now report separate mutation spectra for the older and younger mothers, as well as for all fathers (Supplementary Figure 2). We found absolutely no differences in the mutation spectrum among groups (reported on lines 206-207).

11-	Lastly, what are the variant allele fractions of the DNMs? How many of these are likely post-zygotic mutations? It would be particularly interesting to identify post-zygotic mutations in related trios by looking for shared DNMs between siblings. This could provide insight into whether some of the observed mutations arise post-fertilization.

This is a good point. To address it we have added a new Supplementary Figure 7 that shows the allelic balance for all aye-aye mutations called here, as well as for the mutations transmitted by the mother in the three trios with more maternal mutations. There is no sign at all that these are post-zygotic (lines 249-252).

We mentioned above that there are only two shared mutations (and that it is not clear that they came from through the maternal line). As a further check of post-zygotic mutations and somatic mutations, we assessed the transmission of the mutations we identified in F1 individuals to the F2 generation. Overall, 49% of mutations called were transmitted to the next generation (when it was possible to assess this), which is very close to the 50% expected if these are true germline variants (lines 139-143 and 252-258).

Minor comment: 

Main figures can be improved and benefit from more panel with important information on overall burden between these trios, and other points that are mentioned above.

We hope that the added supplementary figures have provided the information requested.
