# Supplementary material for: Unprecedented female mutation bias in the aye-aye, a highly unusual lemur from Madagascar
Source: PLoS Biol. 2025 Feb 7;23(2):e3003015. doi: 10.1371/journal.pbio.3003015 (PMC11819580; doi:10.1371/journal.pbio.3003015)
Supplement: Attachment — Submitted filename: Responses_v1d.docx [file pbio.3003015.s018.docx]

REVIEWERS' COMMENTS:


Reviewer #1:

Thank you for the revisions and the response to my concerns. Nice work!


Reviewer #2:

The authors have done a nice job with revisions. I only have a couple of minor remaining comments.

The new result on reconciling the phylogenetic estimate of alpha with the de novo mutation data appears to be mentioned for the first time in the discussion. As this is a new analysis, it seems like it should go in the results section about the phylogenetic alpha estimate. I also think this 8.97 years point estimate could benefit from some additional context. Since 8.97 years is earlier than most of the maternal ages in this dataset, I was initially unsure whether an average maternal age this young would be realistic. But when I googled the age of aye-aye puberty, I found that sexual maturity occurs between 2 and 3 years of age, which makes an average reproductive age of 8.97 years seem more reasonable than I initially thought given the age distribution in Figure 2. In addition to providing this biological context, it could be worth putting some kind of rough confidence interval around the 8.97 number. For example, given the lack of a paternal age effect, I infer that any distribution of paternal ages would be compatible with the alpha data; it is only the maternal age distribution that is constrained. It also seems important to note that this 8.97 number comes from a linear interpolation of the maternal age data, which actually looks more bimodal than linear, with basically no maternal age effect if you look at ages <15 years. So it seems unclear whether the maternal mutation load increases linearly versus remains low until age ~15 and then shoots up nonlinearly. Under the second model, the constraint on maternal age would be likely weaker than under a linear model. I don't expect the authors to discuss such possibilities exhaustively, but I would suggest giving some kind of maternal age distribution rather than just a point estimate, and discussing the assumptions and uncertainties associated with the calculation.

As requested, we have now moved this calculation to the Results section (lines 329-342) and have included additional distributions on this age calculation there. The updated text now reads:

“These results reveal a discrepancy between phylogenetic estimates of sex bias and the one estimated from aye-aye pedigrees. Phylogenetic substitution patterns accrue over millions of years, averaging across the age of reproduction of many different individuals during this time. Therefore, if the average age at which aye-ayes reproduced over the past 50 million years is less than ~15 years (Figure 2A), then the major mutation pattern will likely be one of male bias. To get further insight into long-term patterns of sex-bias, we predicted the age of aye-aye parents that would produce the equivalent of the phylogenetic estimate of α=3.1, given our pedigree results (Figure 2A). Assuming male and female parents are the same age at conception—and that sex-bias shows a linear relationship with age—aye-aye parents at an average age of 8.97 years would produce the observed phylogenetic level of male-bias. If we instead assume that the three oldest females sampled here are outliers that can be ignored (for instance, because females do not have offspring at these ages in the wild), then α=2.82, which is within the confidence interval of our phylogenetic estimate (3.1±0.41).”

We have also updated the Discussion to note that the minimum age of aye-aye mothers observed to date is 3.66 years (line 510)

My other minor comment is that the paper's qualitative descriptions of regression strength do not seem consistent. Lines 194-195 talk about a "strong association" with P-value 0.03, while lines 283-284 talk about a "weak association" with P-value 0.04. It might be more appropriate to just describe both associations as "significant" or in some other way that reflects their similar P-values.

We have now changed the former “strong association” to simply “significant association,” and “slight negative association” to simply “negative association.”

COMMENTS FROM THE ACADEMIC EDITOR:

The authors have addressed concerns well but I think they dismiss some arguments too easily and don't fully consider the mutational spectrum. They state in the response that a female bias isn't known in other species and cite humans. But it is known for C->T mutations (Goldmann et al. 2016; Jónsson et al. 2017), as Versoza et al note in the species considered here. Goldman et al for example say:
"...whereas maternally derived DNMs contain more C–T mutations (Bonferroni-corrected P = 2.7 × 10−3, Fig. 3a, Supplementary Table 24)."

They also say:

"Of note, we find an enrichment of maternal DNMs with motifs of APOBEC-mediated mutagenesis (χ2 test for enrichment P = 0.029), which are known to result from aberrant DNA double-strand break repair16. The efficiency of oocyte double-strand break repair is known to decrease in aging women16, which might result in a higher susceptibility to APOBEC-mediated mutations. "

We should clarify that the results cited by the AE above do not reflect a “female bias” in a sense similar to the one reported in our work. Both sentences in Goldmann et al. refer to a higher *proportion* of maternally inherited mutations that are C>T compared to males, relative to all other mutations that are maternally inherited. The total number of such maternally inherited mutations in humans is always much lower than the number that is paternally inherited, at all ages.

I notice too a candidate gene is associated with DSB repair.

That signature is non-CpG mutations - the ones the current authors show are in excess in old females (suppl fig 2). The motif is TpCpN (from the above ref 16).

So a) female bias for some mutations is known, the one they see as being common in older females and b) they should check to see if the female excess is associated with APOBEC TpCN mutations. This all needs to be discussed and related to the prior literature.

Putting aside differences in what is considered female bias, we now report the fraction of maternally inherited mutations that are in a TpCpN context in aye-aye females. We now report (lines 211-226) that neither older mothers and males or older mothers and younger mothers show any difference in the proportion of such mutations:

“Previous studies (Goldmann et al. 2016; Jónsson et al. 2017) have found an enrichment of C>T mutations transmitted by mothers as they age. These excess mutations are likely driven by APOBEC-mediated mutagenesis, which occurs in a TpC dinucleotide context (Alexandrov et al. 2013). To determine whether such an effect is present in aye-ayes, we first compared the proportion of TpC>TpT mutations between male and female parents. We identified 9 TpC>TpT mutations transmitted by a female parent and 5 TpC>TpT mutations transmitted by a male parent, which is not significantly different compared to all C>T mutations that could be assigned to a parent (35 paternal and 33 maternal, excluding those occurring at CpG sites; Fisher's exact test: OR=1.91, *P*=0.38). To further ask whether there was an effect of maternal age, we compared the proportion of TpC>TpT mutations between young (<15 years) and old (>15 years) mothers. Of the 9 TpC>TpT mutations assigned to a female parent, 8 were in old mothers and 1 in a young mother. This distribution is again not significantly different compared to all C>T mutations (28 in old mothers, 5 in young mothers; Fisher's exact test: OR = 1.43, P = 1.0). Importantly, while we may not have the statistical power to detect a subtle effect with maternal age, our results do not support an important role for this mechanism in female mutation bias in aye-ayes.”

Please also update all papers (Young et al is now published)

All references are now completely up to date, as far as we can tell.

Goldmann JM, Wong WS, Pinelli M, Farrah T, Bodian D, Stittrich AB, Glusman G, Vissers LE, Hoischen A, Roach JC, et al. 2016. Parent-of-origin-specific signatures of de novo mutations. Nat Genet. 48(8): 935–939.

Jónsson H, Sulem P, Kehr B, Kristmundsdottir S, Zink F, Hjartarson E, Hardarson MT, Hjorleifsson KE, Eggertsson HP, Gudjonsson SA, et al. 2017. Parental influence on human germline de novo mutations in 1,548 trios from Iceland. Nature. 549(7673): 519– 522.

Versoza CJ, Ehmke EE, Jensen JD, Pfeifer SP. Characterizing the rates and patterns of de novo germline mutations in the aye-aye (Daubentonia madagascariensis). bioRxiv [Preprint]. 2024 Nov 11:2024.11.08.622690. doi: 10.1101/2024.11.08.622690. PMID: 39605388; PMCID: PMC11601268.
